# Using deep learning to assess the toxicological effects of sublethal exposure of a novel green pesticide in a stored‐product beetle

**DOI:** 10.1002/ps.70545

**Published:** 2026-02-03

**Authors:** Anita Casadei, Maria C. Boukouvala, Gianluca Manduca, Nickolas G. Kavallieratos, Filippo Maggi, Marta Ferrati, Eleonora Spinozzi, Cesare Stefanini, Antonio DeSimone, Donato Romano

**Affiliations:** ^1^ The BioRobotics Institute, Sant'Anna School of Advanced Studies Pisa Italy; ^2^ Department of Excellence in Robotics and AI Sant'Anna School of Advanced Studies Pisa Italy; ^3^ Laboratory of Agricultural Zoology and Entomology, Department of Crop Science Agricultural University of Athens Attica Greece; ^4^ ChIP‐Chemistry Interdisciplinary Project Research Center School of Pharmacy, University of Camerino Camerino Italy; ^5^ Department of Robotics Mohamed bin Zayed University of Artificial Intelligence Masdar UAE

**Keywords:** *Prostephanus truncatus*, carlina oxide, natural control, motor behaviour, machine learning

## Abstract

**BACKGROUND:**

Managing stored‐grain pests requires new strategies to limit economic and health risks. This study analyses the sublethal effects of the natural compound carlina oxide on *Prostephanus truncatus*, providing new behavioural insights through a multidisciplinary approach. A fully automatic computer vision approach was developed to label two specific insect body parts, enabling the generation of an annotated dataset without manual intervention. This dataset was used to train a convolutional neural network (CNN) for pose estimation. A second dedicated CNN focused on the antennae to investigate neuroethological and sensory variations.

**RESULTS:**

CNN for body parts detection achieved an average precision of 0.78, recall of 0.90, and F1 score of 0.84 on the test dataset. An additional CNN tracked key points for antennal pose estimation. Motor analysis showed that the LC_30_ of carlina oxide reduced average speed and distance, induced altered exploratory behaviour, and affected thigmotaxis. Statistically significant features were evaluated using machine learning classifiers: random forest, support vector machine, and K‐nearest neighbours. The analysis comparing control and treated groups distinguishes LC_30_ and LC_10_ from the control group, while SHapley Additive exPlanation (SHAP) analysis explained the features contribution to predictions.

**CONCLUSIONS:**

Metrics poorly distinguish individuals in the LC_10_ and LC_30_ classes, supporting the employment of lower sublethal concentration for the control of *P. truncatus*. However, our findings indicate possible neuroethological effects of green pesticides on sensory systems, highlighting the need for an accurate risk assessment to minimize ecosystem impacts and supporting integrated pest management within One‐Health and Eco‐Health frameworks. © 2026 The Author(s). *Pest Management Science* published by John Wiley & Sons Ltd on behalf of Society of Chemical Industry.

## Graphical abstract

This study employs an AI‐driven multidisciplinary approach to increase our understanding of the toxicological effects of sublethal concentrations of carlina oxide on *Prostephanus truncatus*. Sublethal exposure to the carlina oxide induced changes in motor patterns and thigmotaxis, highlighting its potential role in integrated pest management strategies.

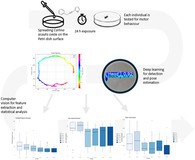

## 1 INTRODUCTION

*Carlina acaulis* L. (Asteraceae) is a perennial herb growing in Central and Southern Europe that is used as a traditional medicine due to its various actions (e.g. diuretic, anti‐inflammatory, and antiicteric) or as food.^1^, ^2^, ^3^, ^4^ The root essential oil (EO) of *C. acaulis* and its main compound (>90%), the polyacetylene carlina oxide, exhibit antibacterial, antifungal, antitrypanosomal, antiproliferative, and antinematode properties.^5^, ^6^, ^7^, ^8^, ^9^, ^10^ They have already been explored as novel green pesticides against several stored‐product pests^11^, ^12^ and field pests such as *Ceratitis capitata* (Wiedemann), *Philaenus spumarius* (L.), the main vector of *Xylella fastidiosa*,^13^, ^14^ and *Metopolophium dirhodum* (Walker).^15^ Interestingly, carlina oxide caused low toxicity to the non‐target species *Aphidoletes aphidimyza* Rondani and *Chrysoperla carnea* Stephens,^15^ an issue that further promotes this substance as green pesticide. Furthermore, Spinozzi *et al*.^16^ documented that carlina oxide did not exert significant toxicity against keratinocytes, while Benelli *et al*.^17^ found that the oral administration of *C. acaulis* EO to Winstar rats at 250 or 500 mg/kg (<LD_50_) did not affect their body weight loss and organs including liver and kidney.

Global losses of stored cereal grains and other agricultural products are estimated at 10–40%,^18^ posing a significant threat to food security and economic stability. Among the most destructive pests is *Prostephanus truncatus* (Horn) (Coleoptera: Bostrichidae), which has spread across multiple countries in the recent decades.^19^ This pest infests stored corn and dried cassava, and without effective inspection and control measures, it poses a risk of global spread.^20^ *Prostephanus truncatus* can also threaten and damage several plant species in forests, stored timber, and related products.^20^, ^21^ The species’ rapid colonization is driven by aggregation pheromones Trunca‐call 1 and Trunca‐call 2,^22^ making *P. truncatus* a critical issue in the context of global food safety and sustainability.^20^

Integrated pest management (IPM) has emerged as a sustainable and multidisciplinary approach to control *P. truncatus* populations while minimizing environmental and health risks.^23^ In this perspective, selecting certain varieties allows the breeding of new crops and enhances the biosynthesis of secondary metabolites active against stored‐product pests, including *P. truncatus*. As shown in literature, the presence of a higher level of phenolic acids in maize can be correlated with a thicker and harder external coat, which could be indirectly useful to avoid the pest progression into the kernels.^24^ IPM strategies also use biological control agents, such as parasitoid wasps (e.g., *Theocolax elegans* (Westwood) and *Pteromalus cerealellae* (Ashmead)) and entomopathogenic fungi (e.g., *Beauveria bassiana* and *Metarhizium brunneum*), which are particularly effective against immature stages (eggs, larvae, and pupae) instead of adults.^23^, ^25^ Additionally, the application of plant‐derived botanicals and EOs leads to reduced impacts on human health and ecosystems.^26^, ^27^ In particular, the EO of *Smyrnium olusatrum* L. inflorescence revealed its effectiveness against this pest because of its high concentration in furanosesquiterpenes like isofuranodiene.^28^

Recently, Boukouvala *et al*.^29^ examined the sublethal effects of a hexane extract of *Acmella oleracea* (L.) R.K. Jansen on *P. trunctatus* mobility, highlighting the reduction of velocity and the increasement of the pest time of stop. However, traditional evaluation methods of pest behaviour often rely on laborious manual observations. To address this gap as well as to improve precision and scalability we propose an innovative precision technology approach that leverages deep learning (DL), machine learning (ML) and computer vision to investigate the motor behaviour of *P. truncatus* when exposed to carlina oxide.

The proposed approach employed a novel computer vision method for the fully automatic labelling of different insect body parts, enabling the creation of a dataset for deep learning‐based pose estimation. A convolutional neural network (CNN) was trained on video recordings to estimate the position of these body parts over time. A second deep learning‐based analysis supported by manual annotation aimed to quantify variations in thigmotactic behaviour and lateralization patterns at the antennal level. A two‐level pose estimation pipeline is proposed, with a first‐stage CNN (20 M parameters) for object detection and classification. This allows rapid and efficient localization of the subject. In the second stage, a ResNet‐50 CNN (25 M parameters) performed fine‐grained pose estimation, enabling antennal tracking for thigmotaxis. By combining these two CNNs, we balance speed and computational efficiency with high‐precision pose estimation.^30^ After both the CNNs application, motor features were extracted from the videos, including speed, distance travelled, arena sector choice, and wall contact across groups. Significant features were used to train classifiers with nested cross‐validation for hyperparameter optimization. Three classifiers, random forest (RF), support vector machine (SVM), and K‐nearest neighbours (KNN), were employed. The classification considers three different binary comparisons followed by SHapley Additive exPlanation (SHAP) analysis to assess features impact on the predicting models. This multidisciplinary framework allows for automated, high‐throughput analysis of behavioural changes that are difficult to observe by naked eye, providing valuable insights into the neuroethological effects of green pesticides. The application of computer vision and ML in pest management provides a novel intersection between bioengineering and environmental management, contributing to food safety and security with minimal environmental impact.^31^

## 2 MATERIALS AND METHODS

### 2.1 *Prostephanus truncatus* rearing

The insects were reared in the Laboratory of Agricultural Zoology and Entomology at the Agricultural University of Athens, under controlled conditions with a constant temperature of 30 °C and 65% relative humidity (RH) as described in Romano *et al*.^32^ For our further trials, we needed separated sexes, so males and females were sexed by focusing on females, which show a wider distance between clypeal tubercles than males.^33^, ^34^

### 2.2 Carlina oxide isolation

Carlina oxide was isolated from *C. acaulis* roots (batch C‐070524‐33) which were acquired from Minardi & Figli S.r.l. (Bagnacavallo, Ravenna, Italy). In detail, the roots were subjected to hydrodistillation following the procedure previously reported by Novák *et al*.,^15^ yielding 0.82% (w/w) of the compound (purity of 95.7% by GC–MS). Its structure was confirmed by NMR and GC–MS analysis and was consistent with that previously reported.^35^

### 2.3 Insects' treatment

The investigation of the sublethal concentrations (i.e. LC_10_, LC_30_, and LC_50_) of carlina oxide against *P. truncatus* was carried out according to the procedure described by Boukouvala *et al*.^29^ Briefly, 30 individuals of *P. truncatus* were exposed to filter papers treated with different concentrations of carlina oxide (i.e. 10 000, 5000, 2500, 1250, 625, 312.5, and 156.25 ppm), and their mortality was recorded after 48 h. Then, we employed LC_10_ and LC_30_ values to expose males and females for 24 h before starting the locomotor assays.^29^ This period of exposure is commonly employed to evaluate lethal or sublethal effects of toxicants, as described in Snell *et al*.,^36^ and it is aligned with the tests previously done considering a period of 48 h to assess the mortality at different concentrations. For this purpose, adults of *P. truncatus* were divided into groups of 32 males and 32 females for each concentration. Two additional groups of untreated *P. truncatus* males and females were used as controls.

### 2.4 Experimental set‐up and video recording

We employed sublethal concentrations, specifically LC_10_ and LC_30_, testing separately 32 valid males and 32 valid females for both concentrations and for control groups. Each group was exposed to carlina oxide for 24 h in a specific Petri dish (Ø = 5 cm). Control groups were collected in two different Petri dishes without carlina oxide exposure. Petri dishes were prepared by placing a disk of filter paper at the bottom of each dish. Subsequently, 1 mL of the corresponding carlina oxide concentration was carefully and homogeneously distributed on the filter paper using a plastic pipette to avoid bias during the analysis.

**Figure 1:**
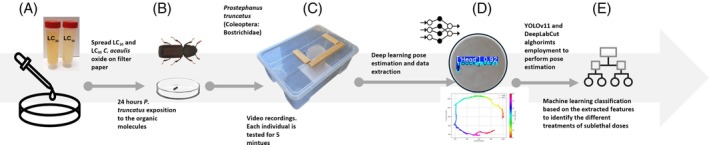
Graphical workflow which merges biological, engineering and AI methodologies to acquire new insight into pest responses to carlina oxide. First, the compound is spread on a Petri dish surface (A), then the individuals are exposed for 24 h, separating males from females (B). Videos are recorded on a customised set‐up (C) and analysed using CNNs (D). Finally, classifiers distinguish control and treated individuals (E).

The experimental setup (Fig. 1) consisted of a plastic box containing a smaller Petri dish, where each individual was tested for behavioural variations. To minimize the possible light exposure induced bias, the structure was enclosed in a paper cylindrical structure. The dish was covered with filter paper to facilitate walking and prevent insects from sliding on the surface. Before recording, each insect was acclimated for 1 min, followed by a 5‐min video recording. The recording time was set in accordance with Manduca *et al*.,^13^ which applied this time for behavioural tests. The setup used an RGB camera (108MP, *f*/1.7) placed over the lid of the box, checking its position before each trial to maintain the same focus. A total of 192 videos were recorded, evenly distributed among the different categories. All recordings were made at a rate of 60 frames per second with a resolution of 1080 × 1920 pixels. All videos were recorded under constant environmental conditions, keeping room temperature around 25 °C and 65% RH. Each individual was tested only once and then replaced with one other individual. After each test the filter paper was replaced to avoid bias in the motorial analysis.

### 2.5 Fully automatic labelling and CNN dataset creation

A fully automated deep learning process was developed to estimate body pose, using 200 frames randomly extracted using OpenCV from six videos across all groups. Each frame was converted to grayscale, blurred using a Gaussian filter (5 × 5 × 5 kernel), and binarized with a threshold value of 60 to enhance contrast and isolate the insect from the background. The overall bounding box of the insect was determined, and progressive erosion was applied iteratively (up to 10 iterations) to separate the insect into two distinct regions corresponding to the head and body. Once these regions were identified, their centroids were calculated and individual bounding boxes were defined for the head and back (dorsum of the body). To differentiate between the head and back, the pixel sizes of the two areas were evaluated, assigning the smaller region to the head and the larger one to the back. With this procedure the body parts were automatically labelled, distinguishing between the anterior part and the posterior one. This approach allowed automatic dataset creation for CNN training, enabling robust and accurate pose estimation across frames. Using a CNN enhances frames generalization, noise robustness, and accurate body parts detection across varying conditions. To train the CNN we employed an image dataset composed of 1200 frames, which was split into three subsets following a 70:20:10 ratio (training, validation, and test), resulting in 840, 240 and 120 images, respectively.

### 2.6 CNN‐based body pose estimation

*Prostephanus truncatus* detection was made using the last released You Only Look Once (YOLO) version. A YOLOv11n model pretrained on a COCO dataset to detect separately two different body parts of the insect. This version has been designed and enhanced to achieve outperforming results in small object detection tasks and a remarkable balance of accuracy and efficiency.^37^ The CNN consists of three main sections: the backbone, neck, and head. A significant improvement in this version is the introduction of the C3k2 block, which employs a smaller kernel to enhance faster training and to allow small size object detection. Moreover, it employs a Cross Stage Partial with Spatial Attention to add precision in tracking spatial disposition of the labels. The CNN can capture multi‐scale information about the analysed images because of its neck section, whose aim is to transmit features to the head for prediction thanks to feature map concatenation. The CNN head is responsible for generating the final predictions of the model. It starts with the feature maps passed by the neck and assigns classes and labels, then draws the respective bounding boxes.^38^

The CNN training process utilized a batch size of eight over 200 epochs. To evaluate the CNN performance precision, recall, accuracy, and F1 score were considered. CNN training was performed using a Tesla T4 graphics processing unit (GPU) available on Google Colab. Video analysis and pose estimation were made suiting the model inference over the entire video dataset. Because of the computationally demanding task, the model was applied to a 2‐min‐long portion of each video isolated in the central part of the original one. This choice is linked to the necessity avoiding possible lag period due to a required acclimation time and to mitigate the possible influence of the testing environment. The inference at video level was performed locally using Ultralytics and Python, and allowed us to obtain the centroid coordinates of the body parts for each frame.

### 2.7 Antennal pose estimation

A more detailed analysis of antennal movement was performed using a computationally heavier CNN, which enabled higher precision for analysing smaller parts that otherwise are difficult to identify. As already shown in literature, DeepLabCut software has demonstrated outstanding reliability, particularly when analysing small species, as in the case of several pests.^39^ The higher resolution makes this software suitable for slight motor behaviours which are not easily observable to the naked eyes. For this reason, Res‐Net 50 CNN was employed to assess variations and lateralization in the thigmotaxis behaviour of the exposed and control *P. truncatus* individuals. Thigmotaxis is considered a behaviour directly connected to anxious condition perception and escape attempts in model species such as zebrafish and mammalian as rodents.^40^ The training dataset consisted of 95% of the total amount of frames while the test dataset of 5% was the standard proposed by DeepLabCut workflow. The selected CNN for antennal pose estimation was a ResNet‐50 CNN trained over 23,500 iterations. Training was stopped early, as no further improvement in performance was observed. This decision was supported by the stabilization of the loss and the learning rate, indicating convergence of the model. After the training we analysed each video of the dataset by applying the trained model. This allowed us to extract both the *x* and *y* positions of the antennae key points as well as the respective value of likelihood. The same two central minutes from each recorded video for thigmotaxis were evaluated. For the antennal CNN, frames were extracted and labelled via a graphical user interface (GUI), and training was conducted on Google Colab using a T4 GPU.

### 2.8 Behavioural feature extraction

Both the CNN models were inferred on the video dataset to provide a pose estimation dataset from which we extracted features such as walking distance, stop time, speed, and acceleration to compile a complete description of the effects of carlina oxide treatments on the motor behaviour of the tested individuals. This analysis was conducted using Python (version 3.10.18), where a low‐pass filter was applied to the velocity data. The filter sets a speed threshold that, according to the literature, is common in several species within the Coleoptera.^41^ We chose to set a threshold to reduce potential bias in the analysis arising from bias during the inference stage. To reduce tracking noise without compromising the motor patterns, an empirical three‐frame rolling window was applied to smooth the body part tracking data. These two inference datasets were essential for studying the trajectories and extracting movement features. The descriptive features considered velocity, covered distance, acceleration, stopping time, number of thigmotactic events for the right and left insects' antennae, number of right and left turns, and the area preference inside the arena. These last features were essential to evaluate possible lateralization in exposed insects. Each analysed feature considered a conversion factor where 1 pixel = 0.055 mm in the experimental set up. Particularly interesting from an ethological and toxicological point of view is thigmotaxis. To count the number of events, the Petri dish was considered as a circumference, whose equation was calculated using the centroids of three non‐collinear points detected using ImageJ software. An event was counted in the thigmotaxis measure when the distance between the centroid of the considered body part and the wall was equal to the radius minus 1 mm of tolerance, similar to Pietri *et al*.^42^ Using the same threshold, wall approaching tendency was quantified as the total time the insect remained in contact with the dish wall. To assess whether the treatment affected the spatial preference, the arena was divided into two zones: an external zone corresponding to the outer circumference (radius = *r*) and an inner zone (radius = 2/3*r*). The time spent in each zone was computed separately. Body orientation was defined as the segment connecting head and back centroids. At each frame, its angle with the frame *x* axis was computed using the *arctg2* function (Eqn 1):

(1) $$\alpha =\arctan2\left(y_{\text{head},i}-y_{\text{back},i},x_{\text{head},i}-x_{\text{back},i}\right)$$

Angles were normalized to [−π, π] to avoid discontinuities. Frame‐to‐frame differences were computed as left (counter clockwise) or right (clockwise), and cumulative rotation was computed by summing them, with each 2π considered one full turn.

### 2.9 Classification models

Statistical analysis was followed by RF, SVM, and KNN based classification of control, LC_10_, and LC_30_ groups using 10 significant motor behaviour features. Results were obtained by employing a nested cross‐validation (CV) using four‐fold external and internal cross‐validation, where the hyperparameter optimization was performed with a grid search. For SVM the *C* values ranged from 0.01 to 100 with ‘rbf’ and ‘linear’ kernels. For RF, optimization was carried out by varying the number of estimators (20, 50, 100, 150, 200, 250, and 300) and the maximum tree depth (2, 3, 4, and 5), while in KNN the number of neighbours (*k*) was tuned over the values 3, 5, 7, 9, 11, 13, and 15. To increase the relevance of our findings, the influence of each analysed feature on the classifier models employing SHAP algorithm was evaluated, which quantifies the contribution of each motor feature to the model's output.^43^

### 2.10 Performance assessment metrics

A reliable and accurate investigation of classifier performances is possible by considering their resulting metrics, which describe the rate of correct classifications made by the algorithm.

Accuracy measures the overall number of correct classifications out of the entire predictions made. It can be computed as:

$$\frac{\mathrm{TP}+\mathrm{TN}}{\mathrm{TP}+\mathrm{TN}+\mathrm{FP}+\mathrm{FN}}$$

where TP denotes the true positives, TN true negatives, FP false positives, and FN false negatives. Recall represents the ratio of correctly predicted positive classifications over all the positive cases in the data. It describes the sensitivity of the model. It can be computed as:

$$\frac{\mathrm{TP}\;}{\mathrm{TP}+\mathrm{FN}}$$

Precision evaluates the proportion of correctly predicted positive observations among all predicted positive instances. It is defined as:

$$\frac{\mathrm{TP}\;}{\mathrm{TP}+\mathrm{FP}}$$

The F1‐score is the harmonic mean of the precision and recall. It can vary from 0 to 1, where a value proximal to 1 represents a well performative model. It is calculated as:

$$F1-\text{score}=\frac{\;2\left(\text{precision}\;\times \;\text{recall}\right)\;}{\left(\text{precision}\;+\;\text{recall}\right)}$$

Intersection over union (IoU) evaluates annotation, segmentation, and object detection accuracy. It quantifies the overlap between predicted and ground‐truth bounding boxes and is calculated as follows:

$$\mathrm{IoU}=\frac{\text{area of overlap}}{\text{area of union}}$$

Mean average precision (mAP) evaluates the CNN performance after training. It corresponds to the mean of the area under the precision–recall curve computed for each class and is defined as:

$$\frac{\mathrm{mAP}=\sum \mathrm{AP}\;\text{for each class}\;}{\text{number of classes}}$$

### 2.11 Statistical analysis

The sublethal concentrations (i.e., LC_10_, LC_30_, and LC_50_) of carlina oxide against *P. truncatus* (95% confidence limits, CL) were calculated using probit analysis^44^ and the statistical software R (version 2.15.1) (R Core Team, R 2017). The motor analysis includes head, back, and body centroid key points pose estimation, sectors preferences, and a further evaluation of lateral differences in antennae thigmotaxis. The collected features were analysed using RStudio software (2024.9.0.375 with 4.4.2 R version). The employed statistical packages were emmeans, ggplot2, glmmTMB, dplyr, and DHARMa. In addition, the statistical analysis accounted not only for the effect of treatments (LC_10_, LC_30_) but also for the interaction between sex and exposure on motor variations. To evaluate the presence of an interaction effect between concentration and sex on the response variable, a generalized linear model was employed. The model (Analysed motor feature ~Sex * Concentration) described the crucial altering effect of the variable concentrations (LC_10_ and LC_30_) (*P* value < 0.001) to stress and the high level of impact of the dosage of carlina oxide on behavioural pattern, but the absence of an effect due to the sex variable. For each feature, a Shapiro–Wilk test was performed to assess the normal distribution. Where the *P* values were below the 0.05 threshold, we applied a Kruskal–Wallis test for each feature, followed by a *post hoc* Dunn test (*P* < 0.05) to investigate statistical significance among groups. Holm's correction was applied as an adjustment for multiple comparisons and to control the FP rate.

## 3 RESULTS

**Figure 2:**
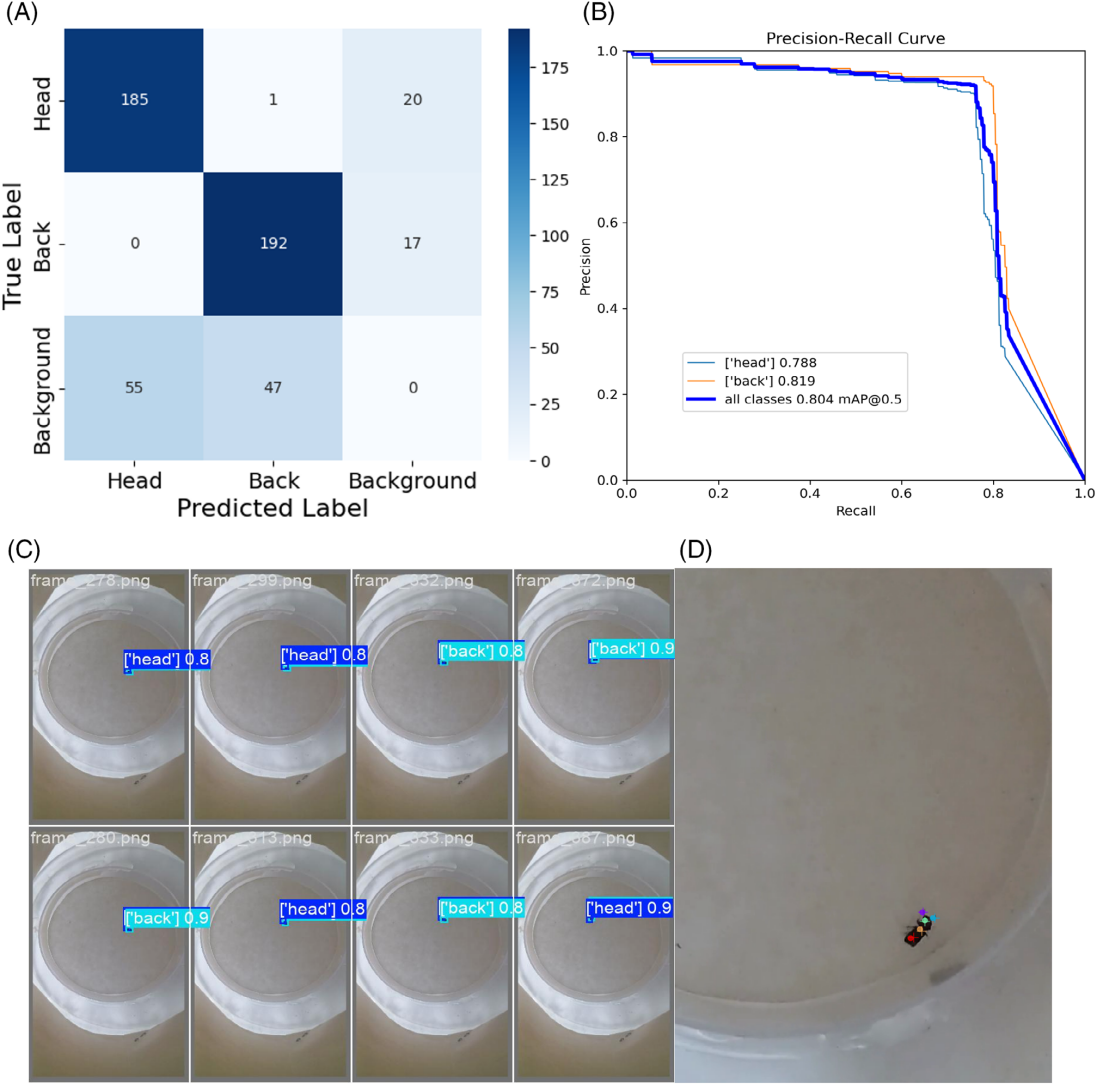
Deep learning results. (A) Confusion matrix of the predicted classes. (B) Precision‐recall curve to assess the CNN performance. (C) Yolov11 test frame, the CNN detects both the classes. (D) DeepLabCut model inference on test frame.

**Figure 3:**
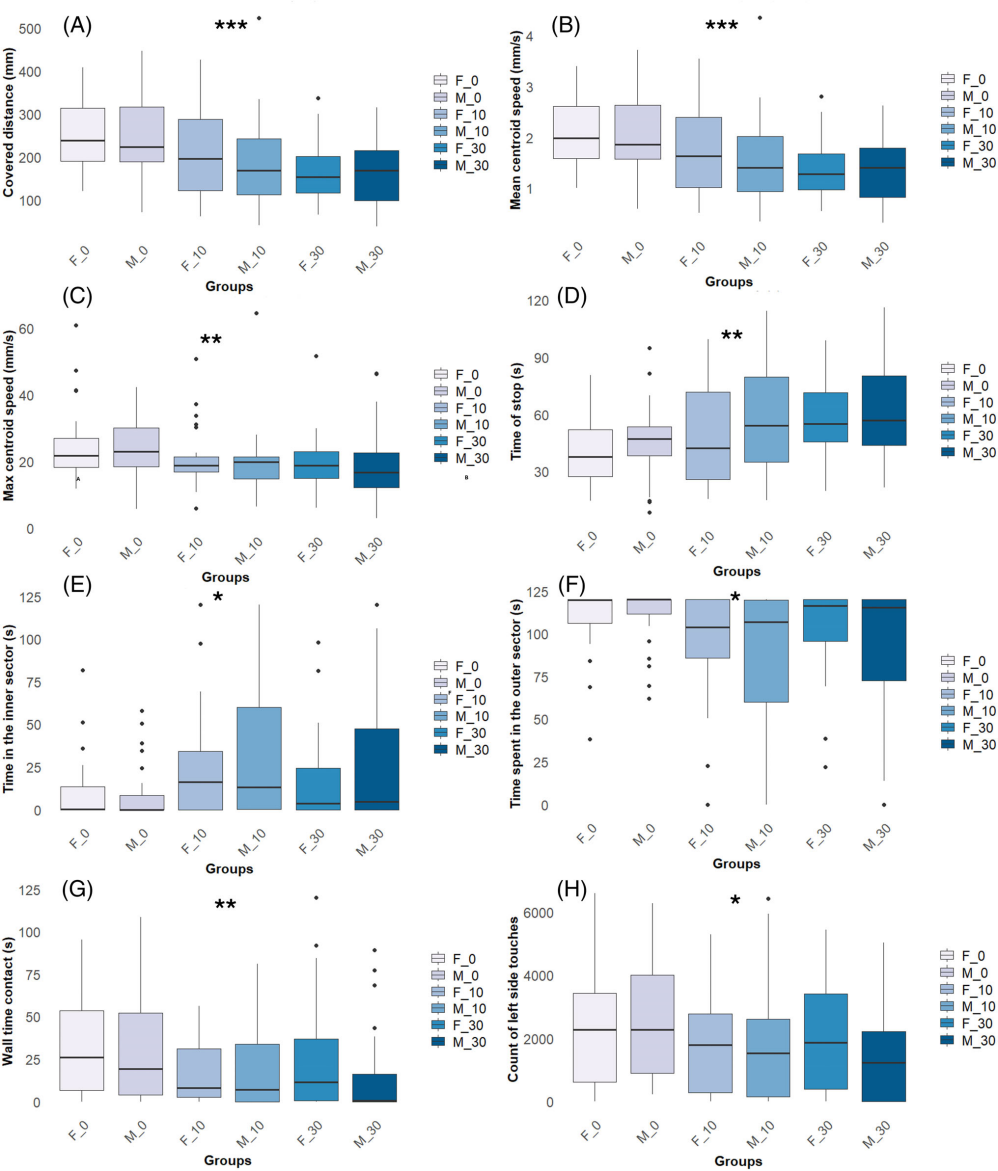
Significant motor behaviours showing sublethal effects on *Prostephanus truncatus* males (M) and females (F): (A) travelled distance, (B) mean speed, (C) maximum speed, (D) time of stop, (Ε) time in the inner sector, (F) time in the outer sector, (G) time in contact with the wall and (H) count of left side thigmotaxis.

**Figure 4:**
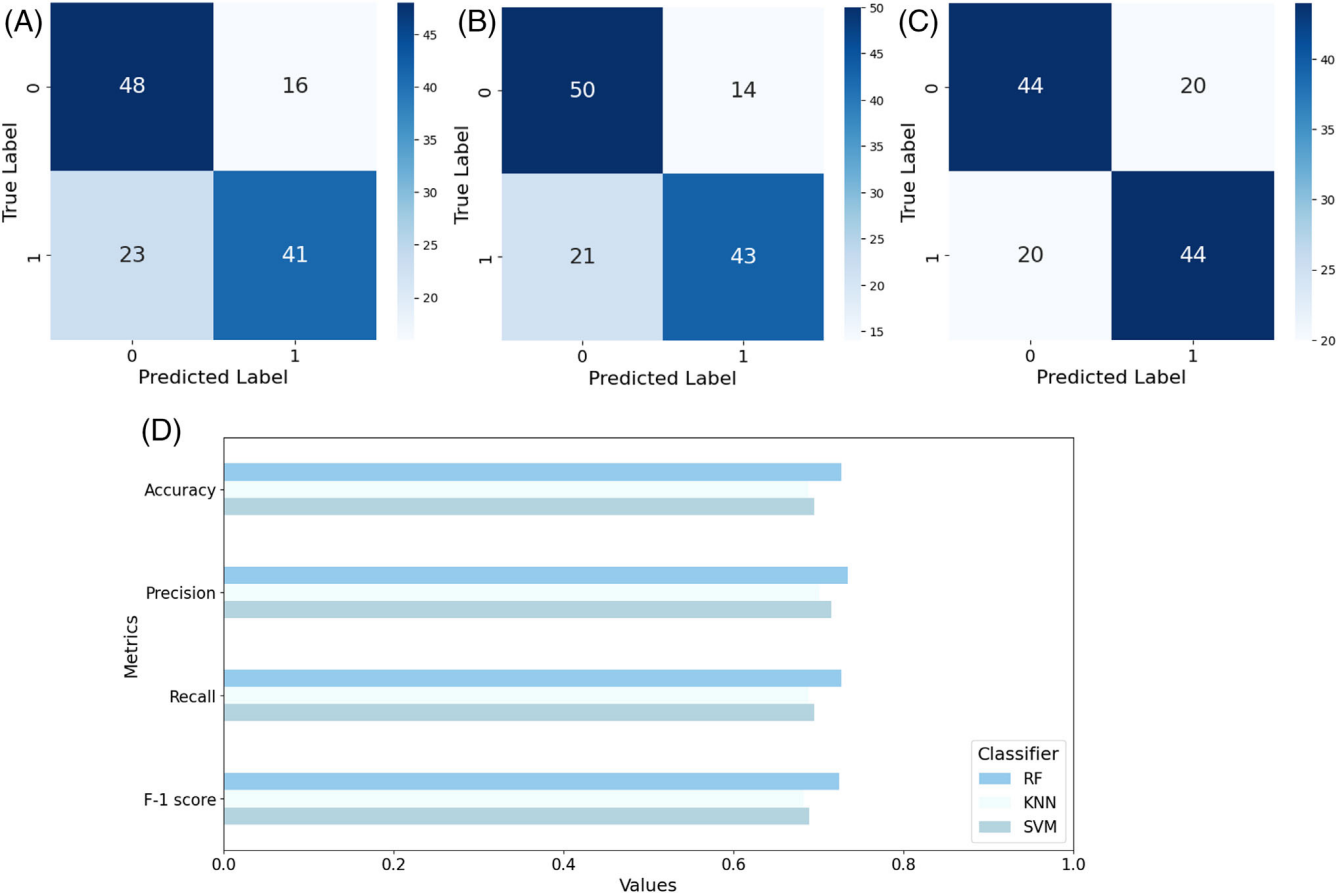
Overall confusion matrix for classifiers comparing control and LC_30_. (A) Support vector machine (SVM), (B) random forest (RF), (C) K‐nearest neighbours (KNN) and (D) metrics barplot for the compared classes.

**Figure 5:**
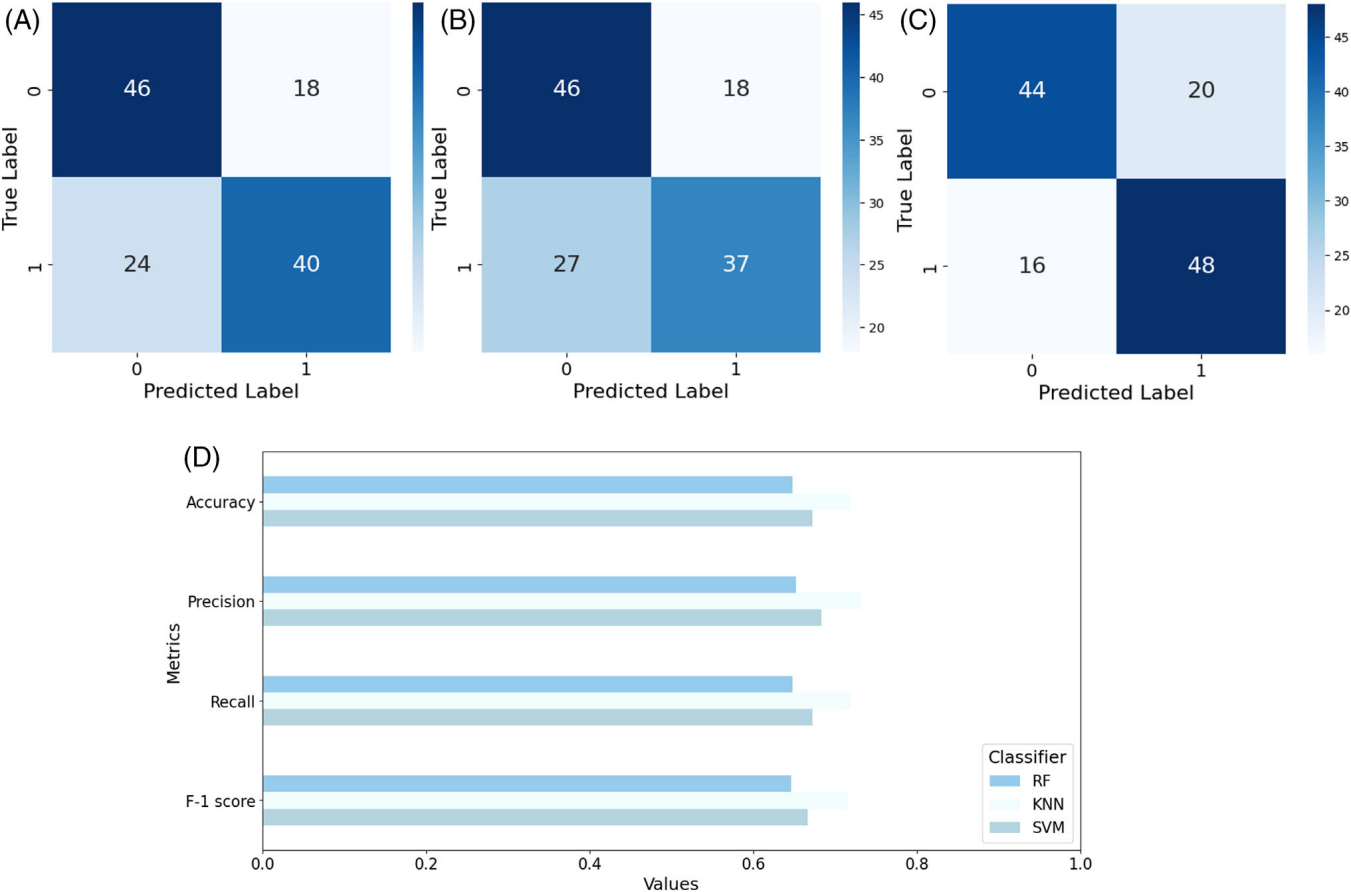
Overall confusion matrix for classifiers comparing control and LC_10_. (A) Support vector machine (SVM), (B) random forest (RF), (C) K‐nearest neighbours (KNN), and (D) metrics barplot for the comparison classes.

**Figure 6:**
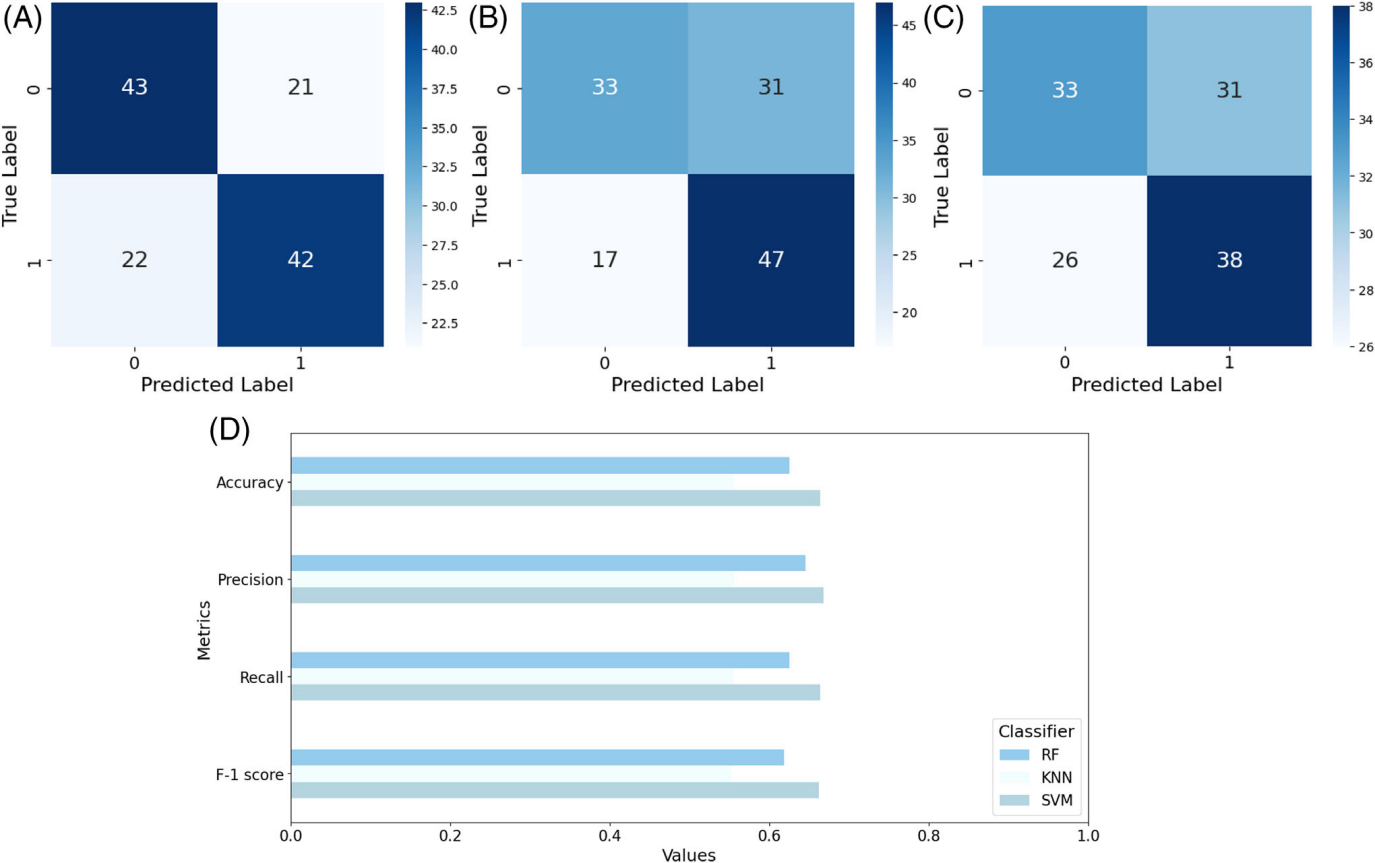
Overall confusion matrix for classifiers comparing LC_10_ class and LC_30_. (A) Support vector machine (SVM), (B) random forest (RF), (C) K‐nearest neighbours (KNN), and (D) metrics barplot for the compared classes.

**Figure 7:**
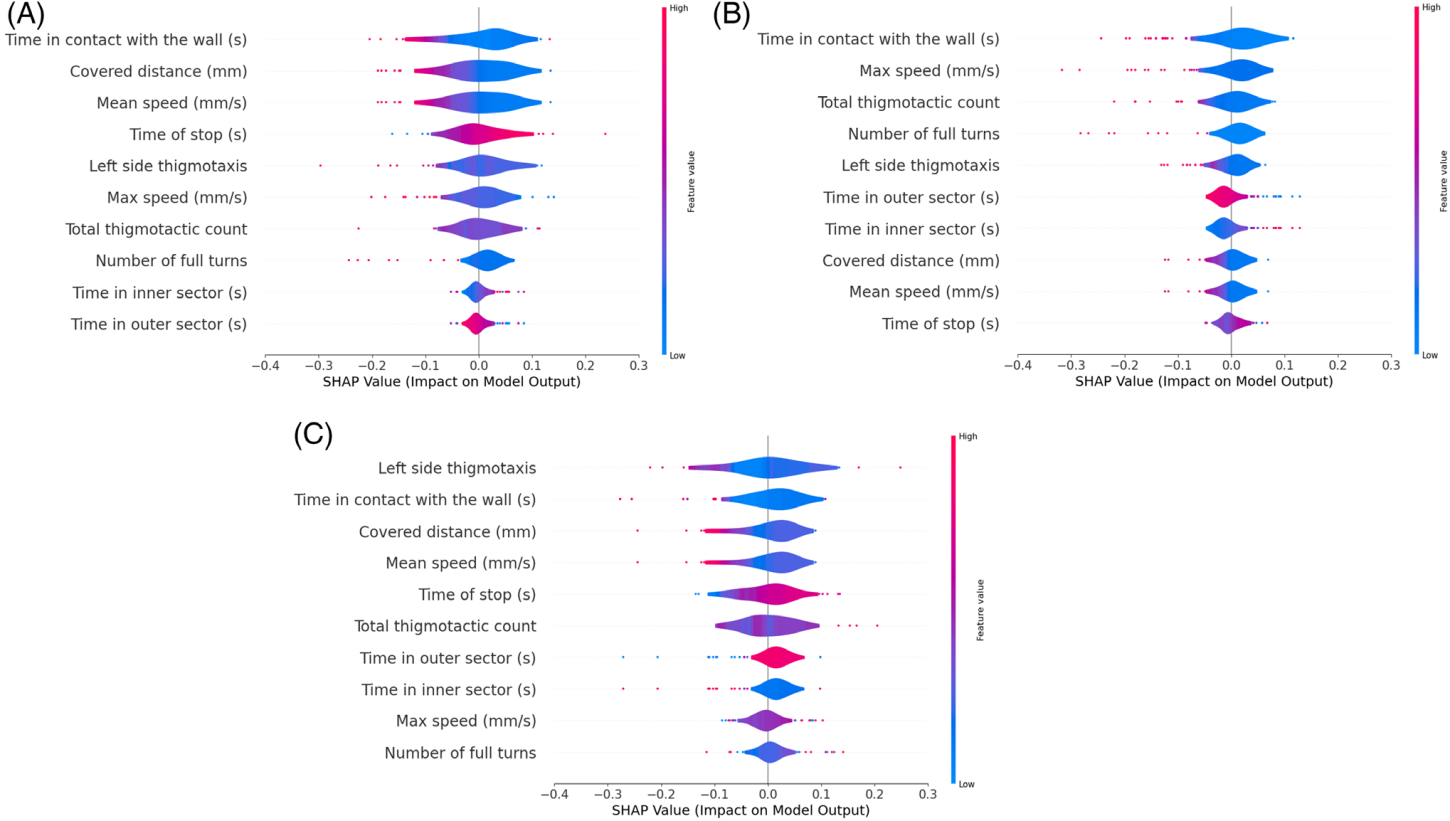
SHAP values of features importance on machine learning model (KNN). Comparisons: (A) control *vs* LC_30_, (B) control *vs* (LC_10_), (C) LC_10_ *vs* LC_30_. Classification relies especially on thigmotactic and spatial preferences.

Concentration‐response tests showed that the carlina oxide LC_10_ was 395 ppm with 95% CL (343–449), LC_30_ was 1028 ppm (936–1122), and LC_50_ was 1992 ppm (1834–2169) (*χ* ^2^ = 114.0, df = 103, *P* = 0.220). The preliminary stereomicroscope observations allowed us to precisely separate male from female individuals considering their sexual clypeal differences. With only naked eye observation, not so obvious variations in motor behaviours can be recognized, despite a reduction in explorative behaviours in *P. truncatus* exposed to LC_30_. The CNN we trained on a costumed dataset of *P. truncatus* frames reached an outstanding level in precision and accuracy, showing a high‐performance level in detecting the body parts. Training the neural network took 2.882 h over 200 epochs using a Tesla T4 GPU. Considering the dataset dimensions, the possibility to complete training in few hours highlights the capabilities of the CNN. The newly obtained model consists of 181 layers, 2 590 230 parameters, and 6.4 GFLOPs of computational power. This number of layers, consisting of convolutional layers, pooling layers, fully connected layers, and activation layers, defines a CNN model able to learn with a high level of accuracy. According to metrics, the model achieved an average precision of 0.78, recall of 0.90, and F1 score of 0.84 as calculated from the confusion matrix (Fig. 2(A)), and a mAP at 0.5 threshold of 0.804. However, the results outlined the higher precision in detecting the back (precision = 80%) bounding box instead of the head (precision = 77%) (Fig. 2(B),(C)). The training outcomes define well‐performing training and a CNN model able to discriminate two different body parts in a small pest, while the possible misclassification is possibly connected to the small size or to the presence of traces of a previously performed test in the set up. The more accurate antennae pose estimation was performed using a Resnet50 CNN. The training was stopped earlier, at 23,500 iterations, to reduce computational demands while maintaining high accuracy and reducing the risk of overfitting. However, it resulted in a highly accurate model with a final loss level of 0.0028 and a learning rate of 0.02. The trained model achieved good accuracy in detecting all key points (Fig. 2(D)), with an average error of 5.22 pixels on the training dataset and 4.51 pixels on the test dataset. The precision levels reached demonstrate the effectiveness of the training process in dealing with a small coleopteran species despite the need to recur to an early stop in training the algorithm. The trained model that was applied to the video dataset determined whether exposure to a potential new green pesticide could influence insects' behaviour. The statistical analysis showed the presence of significative differences in several of the analysed features, such as covered distance, average speed and surprisingly also thigmotaxis. In Fig. 3 is possible to observe the difference between males and females and how this discrepancy is maintained through the different concentration treatments. Here a significative difference was found in left side antennae‐wall tapping (*P* = 0.0241), especially when considering the comparison between the control sex and its treated counterpart. The binary classification, considering one at a time all the possible comparisons among the two exposition concentrations and control, confirmed the statistical analysis. All the classifiers, RF, KNN, and SVM, achieved a good discerning performance when comparing control and LC _30_ (Fig. 4) concentrations, where average precision and accuracy reached 0.72, and 0.70 respectively. Also, classification when comparing control and LC_10_ (Fig. 5), achieved similar results, with an average accuracy among the three classifiers of 0.68, while precision reached 0.69. The misclassification occurred when comparing LC_10_ to LC_30_. Indeed, metrics drop down in this binary comparison (Fig. 6), accuracy was 0.581 and precision 0.595. Moreover, to increase the relevance of our findings, we evaluated the influence of each analysed feature on the classifier models (Fig. 7). This investigation was allowed by SHAP implementation. Our study revealed that treatments significantly reduced locomotion‐related behaviours, including mean and maximum speed and total distance covered. Alterations were also observed in walking area preference and interactions with the Petri dish perimeter (see Fig. 3(F),(G)), both associated with thigmotaxis, which showed a decreasing trend after treatment (Fig. 3(H)).

## 4 DISCUSSION

As shown in the literature, the physiological and behavioural effects caused by the exposure of an individual to toxic compounds have been studied by different methodologies, employing biology, neurobiology, and microscopy techniques.^45^, ^46^ All these approaches, despite their already stated reliability, could be more refined if coupled with informatics and AI applications.^47^ Our findings provide a starting point to deepen knowledge of the effectiveness but also the potentially toxic effect of this novel green pesticide, while also increasing our awareness about pests' ethology and biology.^48^ This research proposes a multidisciplinary approach to study insect behaviour in depth, focusing on patterns of movement and trajectories to demonstrate the effect of a target molecule on pest biology.^49^, ^50^ The possibility of merging biology and AI allowed observer interference to be reduced and provides other innovative tools, including the capacity to record frequency and duration of activities, providing a dataset of activity for subsequent analyses.^51^ The multidisciplinary approach serves as a milestone in the neuroethological investigation allowing to overcome the limitations of naked‐eye‐based techniques. Indeed, this technique introduced issues such as subjective evaluations and other biotic or abiotic variables, which could make human observations less effective than ML and computer vision algorithms.^52^ More in depth, we strengthen the knowledge about the effects deriving from an exposure to sublethal concentrations (LC_10_ and LC_30_) of carlina oxide on *P. truncatu*s adults. We tested the effect related to the interaction between sex and concentration variables on motor behaviour to uncover which lower sublethal concentration can be effectively employed in IPM methodologies. This aspect is particularly relevant because of the growing interest in the medical field of research on gender‐related effects. As discussed by Bonsignore *et al*.^53^ sex‐related effects on metabolism and physiology are common in the survival capabilities of arthropods exposed to a toxicant. In several species belonging to different phyla, females exhibited greater resistance to conventional pesticides such as permethrin and diazinon.^54^, ^55^, ^56^ This may be correlated to a higher esterase activity in females.^57^ Our results confirm the documented sex‐related bias in exposed individuals, particularly in total distance covered and average speed, as shown in group comparisons (Fig. 3(A),(B)). These findings would be particularly difficult to observe without AI application. The employment of YOLO and other DL tools for insect species is not completely new, as shown in Fazzari *et al*.^39^ and also for pest control in livestock barns, as in Santaera *et al*.^58^ However, the constant improvement of the CNN structure allows refinement of the pose estimation with a feasible computational requirement. In this context, the employment of the last YOLO tool version allowed the training process to be enhanced while optimizing the CNN effectiveness and the subsequent general motor analysis,^13^ while the antennal pose estimation results showed variation in thigmotaxis, highlighting a reduction in this behaviour in exposed individuals. This finding is particularly interesting from a neurological point of view because it could mean an increase in boldness caused by exposure to carlina oxide. According to the literature, thigmotaxis is believed to appear as a natural antipredator response or as a response to anxiety, fear, and confusion.^59^ This aspect has been described in different species. For example, a reduction in thigmotaxis has been evaluated in *Danio rerio* larvae which tend to reduce thigmotaxis, described as alert behaviour, when exposed to organophosphate.^59^ This leads to the assumption that some pesticides can cause neurological issues also in non‐target species.^40^ Boldness behaviour, consisting of more time spent wandering in the Petri dish centre in exposed individuals and movement impairments, have been estimated across vertebrate and invertebrate species ranging from fishes to insects, as in *Menidia beryllina* and *Trogoderma granarium* Everts.^60^, ^61^

Our results showed reduced locomotion, including distance, mean, and maximum speed, consistent with Siregar *et al*.^62^ on *Eisenia fetida*, suggesting possible effects on sensory or nervous systems. The influence of carlina oxide on insect motor behaviour has been previously investigated.^13^ The polyacetylene was tested on *C. capitata* at its LC_30_ and five different machine learning algorithms for classification were employed. This revealed low‐concentration carlina oxide effects on medfly locomotion undetected by standard toxicology. The study highlighted that the treated individuals remained motionless compared to the untreated, and simultaneously displayed a higher median speed and less pronounced re‐starts. The effect of carlina oxide seemed to be focused mostly on wing activity, thus indicating a possible influence on the insect central nervous system. However, this hypothesis should be further confirmed.

Moreover, a larger amount of thigmotaxis was negatively correlated in *Gryllus texensis* Cade and Otte with the capability to learn and explore the environment due to a reduction in exposure to visual and olfactory cues.^63^ Although the overall results, when considering the three‐class matrix, are not perfectly precise, the classifiers achieve a good level of performance when considering the three binary comparisons between control and treated individuals. Indeed, the classifiers can discriminate between the control individuals and those exposed to LC_10_ as well as between control individuals and those exposed to LC_30_, with comparable metrics values. Beside this achievement, the classifier accuracy ranged from 0.68 when comparing the control and LC_10_ classes up to 0.70 when comparing the control and LC_30_ classes, showing difficulty in distinguishing individuals subjected to LC_10_ from those exposed to a LC_30_, resulting in misclassification. From a biological perspective, this may lead to positive outcomes in terms of IPM, as it demonstrates that behavioural effects such as boldness and hypoactivity are not strictly significantly different between LC_10_ and LC_30_. This finding suggests that higher concentrations of carlina oxide could be employed in natural control, as previously discussed.^35^ Indeed, these findings are significant when considering the possibility of employing reduced concentrations of a new green pesticide to achieve the same effect on pest control. The impact on both economics and ecotoxicology would be undeniable.

The workflow uses two CNN models: the first one detects individuals at frame and video levels focusing on ‘head’ and ‘back’, and the second estimates antennal poses for detailed behaviour analysis. This approach achieved good results in analysing the locomotory patterns of *P. truncatus*. The classification was supported in relevance with the employment of SHAP with the KNN classifier, which helps in understanding the predictions.^43^ According to the results, when comparing control and LC_30_ groups, speed‐related features, spatial preferences and thigmotaxis values are the most relevant. Low values of this motor features drive the correct classification, instead high values of stop time supporting the classification results. Comparable trends were observed between control and LC_10_ groups, where decreases in thigmotaxis‐related features strongly influenced classification. Overall, the SHAP results were consistent with the statistical analysis and reflected the distribution of the investigated features. Considering the proposed pipeline, some limitations can be discussed to enhance its efficacy in further studies and in wider and multidisciplinary investigation of behavioural patterns of pests. We should consider the absence of an evaluation of the chronic effects. Each *P. truncatus* individual was exposed to carlina oxide for just 24 h. Every possible additional lethal or sublethal effect caused by longer treatments as well as possible delayed effects that could not have been observed with a 5‐min test, should be evaluated in further studies. These effects have been described in several arthropods such as bees, termites, and ants but also freshwater species^64^, ^65^, ^66^ exposed to synthetic pesticides such as neonicotinoids. Moreover, thigmotaxis would be better described in a more articulated arena to avoid possible spatial bias. Despite these limiting aspects, our study combining biology and AI provides an effective and scalable framework to explore the effects of a new green pesticide on *P. truncatus* motor patterns, emphasizing the influence of concentration treatment.

The winning aspect of the followed approach is the possibility to apply it to a wide range of green or synthetic pesticides, obtaining a wider evaluation about their toxicological effects on different species' motor behaviour. It will be particularly important for future toxicological and environmental studies to consider the importance of a One‐Health perspective when dealing with the employment of novel pesticides and their impact on non‐target species. A further step will be evaluating effects across LC_05_–LC_50_ ^67^ to generate descriptive curves for sublethal carlina oxide concentrations. Further developments will aim to deepen the description of lateralization in *P*. *truncatus* and use laboratory assays such as electroantennography to measure the output of an insect antenna for a stimulus. Scanning electron microscopy and transmission electron microscopy applications would significantly advance a more detailed investigation about antennal morphology and ultrastructure.

## CONFLICT OF INTEREST

The authors declare that they have no known competing financial interests or personal relationships that could have appeared to influence the work reported in this article. Mention of trade names or commercial products in this article are solely for the purpose of providing specific information and does not imply recommendation or endorsement by the author institutions.

## Data Availability

The data that support the findings of this study are available from the corresponding author upon reasonable request.
